# First Chemical Constituents from *Cordia exaltata* Lam and Antimicrobial Activity of Two Neolignans

**DOI:** 10.3390/molecules180911086

**Published:** 2013-09-10

**Authors:** Tiago Bezerra de Sá de Sousa Nogueira, Raquel Bezerra de Sá de Sousa Nogueira, Davi Antas e Silva, Josean Fechine Tavares, Edeltrudes de Oliveira Lima, Fillipe de Oliveira Pereira, Milen Maria Magalhães de Souza Fernandes, Fernando Antônio de Medeiros, Rosangela do Socorro Ferreira Rodrigues Sarquis, Raimundo Braz Filho, Jéssica Karina da Silva Maciel, Maria de Fátima Vanderlei de Souza

**Affiliations:** 1 Postgraduate Program in Bioactive Natural and Synthetic Products, Health Sciences Center, Federal University of Paraíba, João Pessoa, PB 58051-970, Brazil; 2 Department of Physiology and Pathology, Health Sciences Center, Federal University of Paraíba, João Pessoa, PB 58051-970, Brazil; 3 Institute for Scientific and Technological Research of Amapá, Macapá, AP 68901-025, Brazil; 4 Laboratório de Ciências Químicas, Universidade Estadual do Norte Fluminense Darcy Ribeiro-UFRRJ, Campos dos Goytacazes, RJ 28013-602, Brazil; 5 Postgraduate Program in Development and Technological Innovation in Drug-Center for Health Sciences, Federal University of Paraíba, João Pessoa, PB 58051-970, Brazil

**Keywords:** phytochemical study, *Cordia exaltata* Lam., Boraginaceae, antimicrobial activity

## Abstract

The phytochemical study of *Cordia exaltata* Lam. (Boraginaceae) led to the isolation, through chromatographic techniques, of nineteen secondary metabolites: 8,8'dimethyl-3,4,3',4'-dimethylenedioxy-7-oxo-2,7'cyclolignan (**1**), 8,8'-dimethyl-4,5-dimethoxy-3',4'-methylenodioxy-7-oxo-2,7'cyclolignan (**2**), sitosterol (**3a**), stigmasterol (**3b**), sitosterol-3-O-β-d-glucopyranoside (**4a**), stigmasterol-3-O-β-d-glucopyranoside (**4b**), phaeophytin **A** (**5**), 13^2^-hydroxyphaeophytin A (**6**), 17^3^-ethoxypheophorbide A (**7**), 13^2^-hydroxy-17^3^-ethoxypheophorbide A (**8**), *m*-methoxy-*p*-hydroxybenzaldehyde (**9**), (*E*)-7-(3,4-dihydroxyphenyl)-7-propenoic acid (**10**), 1-benzopyran-2-one (**11**), 7-hydroxy-1-benzopyran-2-one (**12**), 2,5-bis-(3',4'-methylenedioxiphenyl)-3,4-dimethyltetrahydrofuran (**13**), 3,4,5,3',5'-pentamethoxy-1'-allyl-8.O.4'-neolignan (**14**), 3,5,7,3',4'-pentahydroxyflavonol (**15**),5,7-dihydroxy-4'-methoxyflavone (**16**), 5,8-dihydroxy-7,4’-dimethoxyflavone (**17**), kaempherol 3-O-β-d-glucosyl-6''-α-L-ramnopyranoside (**18**) and kaempherol 3,7-di-O-α-l-ramnopyranoside (**19**). Their structures were identified by ^1^H and ^13^C-NMR using one and two-dimensional techniques. In addition, the antimicrobial activity of compounds **1**, **2**, **13** and **14** against bacteria and fungi are reported here for the first time.

## 1. Introduction

The Boraginaceae family has 148 genera and 2,740 species distributed in the temperate and tropical zones of Europe, Asia, Australia and America [1]. The genus *Cordia*, one of the most representative in the Boraginaceae family [2], consists of approximately 320 species, which are presented as trees, shrubs or herbs, with the main habitat in South America [3]. Its species are used in the folk medicine as diuretics, weight loss agents, healants and emollients [4]. The leaves of the species *Cordia leucocephala*, popularly known as “Maria Preta”, are used as an infusion for the treatment of dysmenorrhea [5]. Biological studies related to the extracts and isolated substances from *Cordia* report antiviral effects against herpes type I, cytotoxic effects on tumor cells CA-9KB [6], anti-inflammatory [7,8] spasmolytic and vasorelaxant [9] properties. The compound 1-(3'-methoxypropanoyl)-2,4,5-trimethoxybenzene, reported as the active principle of *C. alliadora* and commonly found in other species of this genus, was identified as a larvicide for *Aedes aegypti* [10]. Some substances of *C. goetzei* and *C. corymbosa*, are mentioned in the literature for their fungicidal effects against phytopatogens [11,12]. The present study aimed to isolate and identify other classes of secondary metabolites from *C. exaltata* in order to associate them to the observed pharmacological properties. We now report the isolation and identification of four neolignans **1**, **2**, **13** and **14**, two mixtures of steroids **3a**, **3b** and **4a**, **4b**, four porphyrins **5**-**8**, two phenolic acids **9** and **10**, two coumarins **11** and **12** and five flavonoids **15**-**19**. Besides we report for the first time the antimicrobial activity of compounds **1**, **2**, **13** and **14** against bacteria and fungi.

## 2. Results and Discussion

Compound **1** was obtained as colorless crystals. Its IR spectrum exhibited the characteristic absortion of a carbonyl group at 1,678 cm^−1^ and an aromatic nucleus at 1,475 cm^−1^ [13]. The ^1^H- and ^13^C-NMR spectra exhibited the general absortions of a neolignan, with two methyl and two methylenedioxy groups on aromatic rings [14]. The ^1^H-NMR spectrum of **1** exhibited a singlet at δ_H_ 3.80, a multiplet at δ_H_ 2.41 and another multiplet at δ_H_ 1.93, which were attributed to H-7', H-8 and H-8', respectively, while signals at δ_H_ 1.09 and 0.90 were assigned to two methyl groups (H-9 and H-9'). In the aromatic region, an ABX-type system was observed at δ_H_ 6.80 (1H, d, *J* = 8.0 Hz, H-5'), 6.62 (1H, dd, *J* = 8.0 Hz and 1.8 Hz, H-6') and 6.60 (1H, d, *J* = 1.8 Hz, H-2'), attributed to hydrogens of a 1',3',4'-trisubstituted aryl group. Two additional aromatic hydrogens resonate at δ_H_ 7.55 (1H, d, *J* = 8.2 Hz, H-6) and δ_H_ 6.95 (1H, d, *J* = 8.2 Hz, H-5). The ^13^C-NMR spectrum showed 20 carbon resonances, which were assigned by the DPT spectrum as two methyl carbons, three methine carbons, five phenyl methine carbons, two alcoholic methylene carbons, seven phenyl quaternary carbons and a carbonyl carbon. The long-range ^1^H-^13^C-correlations of the HMBC spectrum showing *^3^J* between C-4 (δ_C_ 151.41), C-3 (δ_C_ 144.90) with δ_H_ 5.93 (OCH_2_O-A, 2H, s), C-4' (δ_C_ 145.50), C-3' (δ_C_ 147.00) with δ_H_ 5.83 (1H, s) and δ_H_ 5.78 (1H, s) (OCH_2_O-C), allowed the location of the two methylenedioxy groups to be established and also the assignments of the two methyl positions by way of correlations between C-8 (δ_C_ 42.60) and C-8' (δ_C_ 46.48) with δ_H_ 2.41 (1H, m, H-8) and δ_H_ 1.09 (3H, d, *J* = 6.7 Hz, H-9), respectively, ^2^*J* and ^3^*J*. Additionally, a carbonyl group was located at C-7 by the correlation between the resonances at δ_C_ 197.48 (C-7) and δ_H_ 2.41 (1H, m, ^2^*J*, H-8) and δ_H_ 7.55 (H-6, 1H, d, *J* = 8.7 Hz, ^3^*J*). Thus, compound **1 **was identified as 8,8'-dimethyl-3,4,3',4''-dimethylenedioxy-7-oxo-2,7'-cyclolignan, isolated before from another source [15], but for the first time in genus *Cordia*.

**Figure 1:**
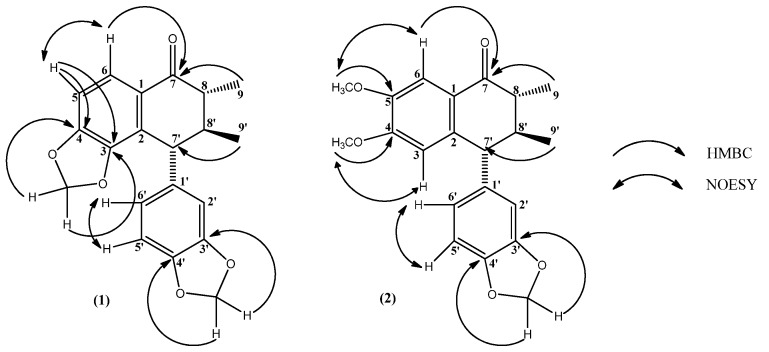
Main HMBC and NOESY interactions for compounds **1** and **2**.

Compound **2** formed white needle-like crystals. Spectral analysis revealed similarities with compound **1**, thus suggesting a neolignan structure for **2** as well. The main difference consisted of two methoxyl groups instead of a methylenedioxy on ring A. This could be inferred by the IR spectrum, which exhibited a C-H absortion from a methoxyl group at 2,850 cm^−1^. The HMBC spectrum showed ^3^*J* correlations between resonances at δ_C_ 149.10 (C-5) and δ_H_ 3.83 (3H, d, *J* = 1.0 Hz, OCH_3_-5), and also δ_C_ 155.06 (C-4) and δ_H_ 3.58 (3H, d, *J* = 1.0, OCH_3_-4), allowing the assignments of the methoxyl groups’ positions. Thus, through a complete spectral analysis, it was possible to identify compound **2**as 8,8'-dimethyl-4,5-dimethoxy-3',4'-methylenodioxy-7-oxo-2,7'-cyclolignan [16], a compound isolated for the first time in the Boraginaceae family. The main correlations of HMBC and NOESY spectra, which allowed a complete assignment for both compounds **1** and **2** are shown in Figure 1.

Compound **13** showed in its ^1^H-NMR spectrum absorptions in the aromatic region, which corresponded to two ABX-systems: at δ_H_ 6.86 (dd, *J* = 8.0 and 1.7 Hz, 2H), at δ_H_ 6.78 (d, *J* = 8.0 Hz, 2H) and another doublet at δ_H_ 6.94 (*J* = 1.7Hz, 2H), suggesting a symmetrical structure. This information was supported by a strong singlet at δ_H_ 5.94, attributed to four hydrogens of two methylenedioxy groups, which is consistent with a neolignan structure. Also, a tetrahydrofuran ring could be inferred fromy two multiplets at δ_H_ 2.24 (2H) and δ_H_ 4.40 (2H), related to H-3/4 and H-2/5, respectively. Two symmetric methyl groups were revealed at δ_H_ 0.99 (*J* = 6.8 Hz, 6H). The ^13^C-NMR spectrum of **13** showed 10 strong peaks, which allowed the identification of two methyl carbons, four methine carbons, two alcoholic methylene carbons, six phenyl methine carbons and six phenyl quaternary carbons, corresponding to 20 chemically equivalent carbons. The HMBC spectrum showed long-range ^1^H-^13^C-correlations between H-2/H-5 (δ_H_ 4.40) and the aromatic carbons at C-6'/C-6' (δ_C_ 120.10) through ^3^*J* and also C-1'/C-1 (δ_C_ 136.16) through ^2^*J*, allowing the location of the two aromatic rings at C-2/C-5. This spectrum could also establish the locations of the two methyl groups, showing ^2^*J* interactions between H-3/H-4 (δ_H_2.24, 2H) and CH_3_-3/CH_3_-4 (δ_C_ 12.97). The ^1^H-^1^H-NOESY spectrum reinforced these positions by showing spatial interactions between H-2/H-5 (δ_H_ 4.40), H-3/H-4 (δ_H_ 2.24) and CH_3_-3/CH_3_-4 (δ_H_ 0.99). Therefore, compound **13** was identified as 2,5-bis-(3',4'-methylenedioxiphenyl)-3,4-dimethyltetrahydrofuran [17], described for the first time in the Boraginaceae family. 

Compound **14** appeared as yellow oil. Its ^1^H-NMR spectrum showed resonances for five methoxyl groups, being two peaks at δ_H_ 3.81 (s, 6H) and δ_H_ 3.77 (s, 6H), which correspond to two pair of symmetric aromatic methoxyls and another group at δ_H_ 3.80 (s, 3H). An allyl group was shown at δ_H_ 5.95 (ddt, *J* = 17.0, 10.5 and 6.5 Hz, 1H) (H-8'), δ_H_ 5.09 (ddt, *J* = 10.5, 2.0 and 1.6 Hz, 1H, H-9'a), δ_H_ 5.05 (ddt, *J* = 17.0, 2.0, e 1.6 Hz, 1H, H-9'b) and δ_H_ 3.31 (d, *J* = 6.5 Hz, 2H, H-7'). Additionally, two singlets were observed at δ_H_ 6.44 (2H) and δ_H_ 6.38 (2H), referring to two pairs of symmetric phenyl hydrogens, H-2/H-6 and H-2'/H-6', respectively. From these absorptions it was possible to suggest an aryloxyarylpropane structure for compound **14**, consistent with a neolignan skeleton. Through HMBC analysis, the methoxyl groups positions could be assigned by ^3^*J* ^1^H-^13^C correlations showed between resonances at δ_H_ 3.81 (H_3_CO-3/5) with δ_C_ 153.64 (C-3/5) and δ_H_ 3.77 (H_3_CO-3'/5') with δ_C_ 152.85 (C-3'/5'). Also, hydrogen H-7' (δ_H_ 3.31, *J* = 6.5 Hz, 2H) showed ^3^*J* and ^2^*J* correlations with C-2'/6' (δ_C_ 105.94) and C-9' (δ_C_ 115.94), respectively, leading to the location of the allyl group at C-1'. Therefore, compound **14** was identified as 3,4,5,3',5'-pentamethoxy-1'-allyl-8.O.4'-neolignan [17], described here for the first time in the Boraginaceae family.

**Figure 2:**
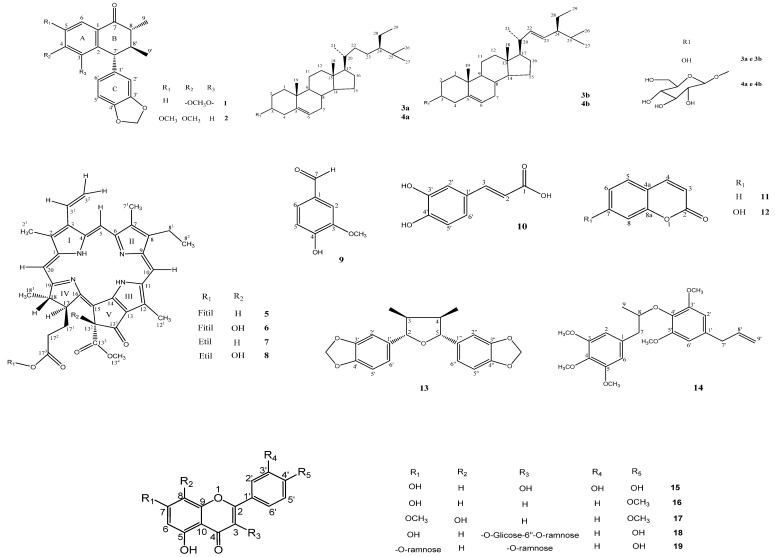
Constituents isolated from *Cordia exaltata*.

The spectral analysis using IR and ^1^H/^13^C-NMR data by means of one and two-dimensional techniques was also performed for the other compounds, which made possible their identification as sitosterol (**3a**), stigmasterol (**3b**), sitosterol-3-*O*-β-d-glucopyranoside [18] (**4a**), stigmasterol-3-*O*-β-d-glucopyranoside (**4b**) [18], phaeophytin A (**5**) [19], 13^2^-hydroxyphaeophytin A (**6**) [20], 17^3^-ethoxypheophorbide A (**7**) [21], 13^2^-hydroxy-17^3^-ethoxypheophorbide A (**8**) [22], *m*-methoxy-*p*-hydroxy-benzaldehyde (**9**) [23], (E)-7-(3, 4-dihydroxyphenyl)-7-propenoic acid (**10**) [24], 1-benzopyran-2-one (**11**) [25], 7-hydroxy-1-benzopyran-2-one (**12**) [26], 3,5,7,3',4'–pentahydroxyflavone (**15**) [27], 5,7-dihydroxy-4'-methoxyflavone (**16**) [27], 5,8-dihydroxy-7,4'-dimethoxyflavone (**17**) [28], kaempferol 3-*O*-β-d-glucosyl-6''-α-L-ramnopyranoside (**18**) [29], and kaempferol 3,7-di-*O*-α-l-ramnopyranoside (**19**) [30] (Figure 2).

The MIC values of compound **1** are shown in Table 1. As can be seen, 90% of the strains were susceptible to the compound up to the concentration of 300 µg/mL, and the percentage of inhibition was higher than that observed with the drug controls—chloramphenicol for bacteria and ketoconazole for yeasts—where 60% of strains where inhibited by the respective antibiotics. The MIC of the compound **1** was 150 µg/mL to almost all strains tested, where about 75% of the strains had their growth inhibited by this concentration. It was observed that compound **1** showed the greatest inhibiting effect on strains of *C. guilliermondii* LM-2101 and *C. guilliermondii* LM-011, with showed the lowest MIC values (75 µg/mL).

The MIC data of the compound **2** are also summarized in Table 1. It was observed that the MIC was 300 for 35% of the strains. The percentage of strains resistant to the compound **2** to the highest concentration was 40%, therefore higher than that found with the compound **1** (10%). As for the antibiotics tested, they showed inhibitory effect on 60% of the strains, similar to compound **2**.

Thus, one can conclude that both the tested compounds have detectable antimicrobial activity against a variety of pathogens, including bacteria and yeasts, in infectious processes relevant from a clinical point of view. It also may be noted the compound **1 **had the strongest antimicrobial effect, which could be explained by the lower MIC values against microorganisms [31,32,33,34]. Compounds **13 **and **14** were also tested against the same strains and following the same protocol described for **1** and **2**, but did not show any significant activity.

**Table 1:** MIC values of compounds **1** and **2** against bacteria and yeasts.

Microorganisms	(1) (µg/mL)	(2) (µg/mL)	Controls		
			Tween 80	Microrganisms	Antimicrobyal
*S. aureus* ATCC-6538	150	>300	+	+	-
*S. aureus* ATCC-25923	150	>300	+	+	+
*S. epidermidis* ATCC-12228	150	>300	+	+	-
*B. subtilis* ATCC-6633	150	>300	+	+	-
*P. aeruginosa* ATCC-25853	150	300	+	+	+
*P. aeruginosa* ATCC-9027	150	300	+	+	+
*E. coli* (clássica C)	150	300	+	+	-
*E. coli* ATCC-18739	150	300	+	+	+
*E. coli* ATCC-8733	150	>300	+	+	+
*S. flexineri* LM-412	150	150	+	+	-
*C. albicans* ATCC-90028	150	150	+	+	-
*C. albicans* ATCC-76615	150	150	+	+	-
*C. albicans* LM-142 V	150	75	+	+	-
*C. albicans* ICB-12	150	300	+	+	-
*C. tropicalis* ATCC-13803	>300	300	+	+	+
*C. tropicalis* LM-028	>300	>300	+	+	-
*C. krusei* ATCC-6258	150	75	+	+	+
*C. krusei* LM-12	300	300	+	+	-
*C. guilliermondii* LM-2101	75	>300	+	+	-
*C. guilliermondii* LM-011	75	>300	+	+	+

+: Presence of microbial growth; −: Absence de microbial growth.

## 3. Experimental

### 3.1. General

Silica gel 60 (Merck) 7734 (0.063–0.2 mm particle, 70–230 mesh), flash silica (0.04–0.063 mm particles, 230–400 mesh) and Sephadex LH-20 were used for the fractionation and isolation of the secondary metabolites from *C. exaltata*. TLC was used to analyse and compare the fractions obtained from chromatographic column procedures. Solvents used in TLC were basically mixtures of the same solvents of the chromatographic experiments. For pure compounds analysed by TLC, three different systems of solvents were tested, to make sure that these compounds were free of impurities. The melting point of the constituents was recorded on a MQAPF-302 apparatus. IR spectra were recorded on a FT-IR-1750 Perkin-Elmer spectrometer. ^1^H and ^13^C-NMR spectra were recorded on a Varian Oxford 200 NMR spectrometer (200/50 MHz) and on a Varian 500 NMR spectrometer (500/125 MHz).

### 3.2. Collection, Extraction and Isolation

The leaves, stems, fruits and stem bark of *Cordia exaltata* Lam, were collected near the city of Porto Grande–AP in July 2006 and were identified by Prof. Dr. Rosangela do Socorro Ferreira Rodrigues Sarquis-IEPA (Instituto de Pesquisa Científica e Tecnológica do Estado do Amapá/BR-Institute for Scientific and Technological Research of Amapá). A voucher specimen is deposited in the Herbarium Amapaense/AP/BR (HAMAP), under the code 2528.

Leaves, stems, fruits and stem bark of *Cordia exaltata* Lam were dehydrated in an oven at 40 °C for 72 h and ground separately in a mechanical mill to yield 847.5 g, 1000.5 g, 281.0 g and 2.310.0 g of a powder, respectively. Each powdered part of the plant was submitted to maceration with methanol (2.5 L, 3.0 L, 1.0 L and 4 L, respectively) for three consecutive days at room temperature and this process was repeated until the maximum amount of chemical constituents had been extracted. The obtained methanol extract solutions were concentrated in a rotatory evaporator, yielding 150.0 g, 150.0 g, 11.0 g and 211.0 g of the respective crude extracts.

An amount of the crude methanolic extract from fruits (12.0 g) was subjected to filtration under reduced pressure using silica gel 60 as stationary phase and eluted with hexane (hex.), ethyl acetate (EtOAc) and methanol (MeOH) alone or in binary mixtures following an increasing gradient polarity. After that, the extracts were concentrated under pressure, leading to the respective phases. The hexane phase (1695.0 mg) yielded 847.8 mg of a precipitate, which was chromatographed on a silica gel column and eluted with hex., EtOAc and MeOH. This procedure led to 28 fractions, analysed by TLC (hexane, EtOAc and MeOH at least in three different polarities), from which the combined fractions 5/14 (200.0 mg) and 17/20 (170.0 mg) gave compounds **1 **and **2**, respectively.

The methanolic crude extract from the leaves of *C. exaltata* Lam. (100.0 g) was suspended in methanol-H_2_O (9:1) and successively partitioned with hex., chloroform (CHCl_3_), EtOAc and *n*-butanol, increasing the gradient polarity. The hexane phase (10.0 g) was chromatographed under the same conditions as described previously for the crude extract of fruits, giving 80 fractions, which were combined by TLC (hexane, EtOAc and MeOH mixtures of at least three different polarities). Fractions 1/19, after purification, led to 100.0 mg of a mixture of compounds **3a** and **3b**. Fraction 20/27 was chromatographed on a gel silica column, from which 84 fractions were obtained and analysed by TLC. From this chromatography, fractions 01/08, 09/18 and 28/36 were purified and this led to the isolation of compounds **4** (40 mg), **5** (100.0 mg) and **6** (100.0 mg), respectively. The CHCl_3_ extract (8.18 g) was also chromatographed following the described methodology, which allowed the isolation and purification of 30.0 mg of compound **11**, 35.0 mg of compound **12** and 17.0 mg of **16**. The EtOAc (3.7 g) and *n*-butanol (1.4 g) phases were submitted to chromatography on Sephadex LH-20, affording compound **15** (40.0 mg) and **19** (15.0 mg), respectively.

The methanolic crude extract from the stem bark (100 g) was solubilized in methanol-H_2_O (7:3) and successively partitioned under vacuum using silica gel with hex., CHCl_3_, EtOAc and *n*-butanol as described in the previous procedures. The hexane phase (5.0 g) was submitted to column chromatography with silica gel using hexane (hex.), ethyl acetate (EtOAc) and methanol (MeOH) alone or in binary mixtures with increasing polarity, leading to 93 fractions, which were further combined according to TLC (hex., AcOEt and MeOH, in three different mixtures at least). Using this technique fractions 16/24, 37/47 and 57/70 were pure and gave 25.0 mg of compound **8**, 18.0 mg of compound **9** and 40.0 mg of compound **10**. Through column chromatography on Sephadex LH-20, the EtOAc (2.9 g) and *n*-butanol (2.6) phases yielded compound **17 **(15.0 mg) and compound **18** (30.0 mg), respectively.

The bark extract of *C. exaltata* Lam (7.5 g) was chromatographed on silica gel under reduced pressure with the following solvents: hex., hex-EtOAc (9:1), hex.-EtOAc (6:4), hex.-EtOAc (1:1), Hex.-EtOAc (4:6), AcOEt, EtOAc-MeOH (9:1) and EtOAc-MeOH (1:1). Fraction Hex.:AcOEt (6:4) (175.0 mg) was fractionated by column using gel silica and an increasing polarity system of solvents, such as Hex., EtOAc and MeOH, which result in 30 fractions. These fractions were analysed by TLC, using Hex.-EtOAc in three different mixtures of polarity. Fractions 02/04 and 08/11 were purified and led to the isolation of compound **13** (10.0 mg) and compound **14** (12.0 mg), respectively. The fraction eluted with EtOAc-MeOH (9:1) yield a supernatant and a precipitate. The latter was separated from the former, purified and analysed by TLC using EtOH-MeOH (9:1) as eluting solvent, which resulted in a mixture of compounds **4a** and **4b **(30 mg).

### 3.3. Spectral Data

*8.8'-Dimethyl-3,4,3', 4'-dimethylenedioxy-7-oxo-2,7'-cyclolignan* (**1**). Colorless crystals; mp 176 °C; IR (KBr) v_máx_ (cm^−1^): 3081, 2963, 1678 (C=O), 1475; ^1^H-NMR (D_­_MSO-d_6_, 500 MHz): δ 7.55 (1H, d, *J* = 8.2 Hz, H-6), 6.95 (1H, d, *J* = 8.2 Hz, H-5), 6.80 (1H, d, *J* = 7.9 Hz, H-5'), 6.62 (1H, dd, *J* = 8.0 Hz e 2.0 Hz, H-6'), 6.60 (1H, d, *J* = 1.7 Hz, H-2'), 5.93 (2H, s, OCH_2_O-A), 5.83 (2H, s, OCH_2_O-C), 3,80 (1H, d, *J* = 9.8 Hz, H-7'), 2.41 (m, H-8), 1.09 (1H, d, *J* = 6.7 Hz, H-9), 1.93 (m, H-8'), 0.90 (1H, d, *J* = 6.7 Hz, H-9'); ^13^C- NMR (D_­_MSO-d_6_, 125 MHz): δ 197.48 (C-7), 151.41(C-4), 147.00 (C-3'), 145.50 (C-4), 144.90 (C-3), 137.50 (C-1'), 126.84 (C-2), 122.05 (C-6), 121.83 (C-6'), 108.67 (C-2'), 107.82 (C-5'), 107.46 (C-5), 100.72 (OCH_2_O-A), 101.54 (OCH_2_O-C), 47.97 (C-7'), 46.48 (C-8'), 42.60 (C-8), 17.11 (C-9), 12.45 (C-9').

*8,8'-Dimethyl-4,5-dimethoxy-3',4'-methylenedioxy-7-oxo-2,7'cyclelignan* (**2**). Fine white crystals; mp 131 °C; IR (KBr) v_máx_ (cm^−1^): 3081, 2963, 2850 (OCH_3_), 1678 (C=O), 1475; ^1^H-NMR (CD_3_OD, 500 MHz): δ_H_ 7.47 (1H, d, *J* = 1.0 Hz, H-6), 6.24 (1H, sl, H-3), 6.81 (1H, dd, *J* = 8.0 Hz and 2.0 Hz, H-6'), 6.70 (1H, d, *J* = 8.0 Hz, H-5'), 6.59 (1H, sl, H-2'), 5.94 (2H, s, OCH_2_O-C), 3.83 (3H, d, *J* = 1.0, OCH_3_-5), 3.74 (1H, d, *J* = 11.50 Hz, H-7'), 3.58 (3H, d, *J* = 1.0, OCH_3_-4), 2.39 (1H, dq, *J* = 13.0 Hz and 6.5, H-8), 2.04 (1H, ddq, *J* = 12.5, 11.5 and 6.0 Hz, H-8'), 1.26 (1H, dd, *J* = 6.5 and 0.5 Hz, H-9), 0.93 (1H, dd, *J* = 6.5 and 1.0 Hz, H-9'); ^13^C-NMR (CD_3_OD, 125 MHz): δ_C_ 200.79 (C-7), 155.06 (C-4), 149.49 (C-3'), 149.10 (C-5), 143.51 (C-2), 138.89 (C-1'), 126.68 (C-1), 124.20 (C-5'), 112.57 (C-3), 110.14 (C-2'), 109.42 (C-6), 109.10 (C-6'), 102.40 (OCH_2_O-C), 56.17 (OCH_3_-4), 54.28 (C-7'), 49.52 (C-8), 44.98 (C-8'), 18.17 (C-9'), 12.87 (C-9).

*Sitosterol* (**3a**) *and stigmasterol* (**3b**). ^1^H-NMR and ^13^C-NMR data were consistent with the literature values [17].

*Sitosterol-3-O-β-d-glucopyranoside* (**4a**) and *stigmasterol-3-O-β-d-glucopyranoside* (**4b**). ^1^H-NMR and ^13^C-NMR data were in agreement with the literature [17].

*Phaeophytin A* (**5**). IR, ^1^H-NMR and ^13^C-NMR data were in agreement with the literature [21].

*13^2^-Hydroxyphaeophytin A* (**6**) (without phytyl ester). Green powder. IR (KBr) vmax: 3494, 2926, 2854, 1739, 1706, 1620, 1377 cm−1. ^1^H-NMR and ^13^C-NMR data were in agreement with the literature [35].

*17^3^-Ethoxyphaeophorbide A* (**7**). ^1^H-NMR and ^13^C-NMR data were in agreement with the literature [21].

*13^2^-Hydroxy-17^3^-ethoxyphaeophorbide A* (**8**). Bluish green powder with a metallic luster. ^1^H-NMR and ^13^C-NMR data were in agreement with the literature [22].

*m-Methoxy-p-hydroxybenzaldehyde* (**9**). White crystals; mp 81 °C–82 °C. ^1^H-NMR and ^13^C-NMR data were in agreement with the literature [23].

*Kaempferol 3,7-di-O-α-L-ramnopyranoside* (**19**). Yellow crystals; mp 201 °C–203 °C. ^1^H-NMR and ^13^C-NMR data were in agreement with the literature [36].

*(E)-7-(3,4-Dihydroxyphenyl)-7-propenoic acid* (**10**). White solid; mp 220–221 °C. ^1^H-NMR (CD_3_OD, 200 MHz): δ_H_7.40 (1H, d, *J* = 15.9 Hz, H-7), 6.08 (1H, d, *J* = 15.9 Hz, H-8), 6.91 (1H, d, *J* = 1.9 Hz, H-2), 6.79 (1H, dd, *J* = 8.2, 1.9, Hz, H-6), 6.64 (1H, d, *J* = 8.2 Hz, H-5); ^13^C-NMR (CD_3_OD, 50 MHz): δ_C_127.71 (C-1), 115.43 (C-2), 116.45 (C-5), 122.88 (C-6), 149.35(C-4), 146.66 (C-3), 171.12 (C-9), 114.98 (C-8), 147.04 (C-7).

*1-Benzopyran-2-one* (**11**). White solid, mp 69–70 °C. ^1^H-NMR (CDCl_3_, 200 MHz): δ_H_ 7.52 (1H, d, *J* = 9.6 Hz, H-3), 6.22 (1H, d, *J* = 9.6 Hz, H-4), 7.12 (1H, dd, *J* = 7.0 and 1,0 Hz, H-8), 7.06 (1H, dd, *J* = 7.86, 6.4 and 1.0 Hz, H-6), 7.35 (1H, dd, *J* = 7.86, 6.4 and 1.25 Hz, H-7) 7.30 (1H, dd, *J* = 6.4 and 1.25 Hz, H-5); ^13^C-NMR (CDCl_3_, 50 MHz): δ_C_ 160.89 (C-2), 116.96 (C-3), 116.76 (C-8), 118.90 (C-4a), 154.10 (C-8a), 143.57 (C-4), 131.93 (C-7), 127,97 (C-5), 124.53 (C-6).

*7-Hydroxy-1-benzopiran-2-one* (**12**). White solid; mp 230–231 °C.^1^H-NMR (CD_3_OD, 500 MHz): δ_H_ 7.87 (1H, d, *J* = 9.4 Hz, H-4), 6.22 (1H, d, *J* = 9.4 Hz, H-3), 7.48 (1H, d, *J* = 8.5 Hz, H-5), 6.83 (1H, dd, *J* = 8.5 and 1.9 Hz, H-6), 6.75 (1H, s, H-8); ^13^C-NMR (CD_3_OD, 125 MHz): δ_C_ 163.66 (C-2), 112.34 (C-3), 103.41 (C-8), 113.49 (C-4a), 157.21 (C-8a), 145.99 (C-4), 163.09 (C-7), 130,62 (C-5), 114.49 (C-6).

*2,5-bis-(3',4'-Methylenedioxyphyenyl)-3,4-dimethyltetrahydrofurane* (**13**). ^1^H-NMR (CD_­_Cl_3_, 500 MHz): δ_H_ 6.94 (2H, d, *J* = 1.7 Hz, H-2' and 2''), 6.86 (2H, d, *J* = 8.0 and 1.7 Hz, H-6' and 6''), 6.78 (2H, d, *J* = 8.0 Hz, H-5' and 5''), 5.94 (4H, s, H-OCH_2_O-), 4.40 (2H, d, *J* = 6.8 Hz, H-2 and 5), 2.24 (2H, m, H-3 and H-6) and 0.99 (6H, d, *J* = 6.8 Hz, H-CH_3_-3 and CH_3_-4), ^13^C-NMR (CD_­_Cl_3_, 125 MHz): δ_C_ 147.92 (C-3' and 3''), 147.14 (C-4' and 4''), 136. 16 (C-1' and 1''), 120.10 (C-6' and C-6''), 108.15 (C-5' and 5''), 106.91 (C-2' and 2''), 101.09 (-OCH_2_O-), 87.59 (C-2 and 5), 44.80 (C-3 and C-4), 12.97 (C-CH_3_-3 and CH_3_-4).

*3,4,5,3',5'-Pentamethoxy-1'-allyl-8.O.4'-neolignan* (**14**). ^1^H-NMR (CDCl_3_, 500 MHz): δ_H_ 6.44 (2H, s, H-2 and 6), 6.38 (2H, s, H-2' and 6'), 5.95 (1H, ddt, *J* = 17.0 Hz, 10.0 Hz and 6.5 Hz, H-8), 5'.09 (1H, ddt, *J*=10.5 Hz, 2.0 Hz and 1.6 Hz, H-9'a) 5,05 (1H, ddt, *J* = 17.0 Hz, 2.0 Hz and 1.6 Hz, H-9'b), 4.33 (1H, dt, *J*=7.6 Hz and 5.3 Hz, H-8), 3.81 (6H, s, H- H_3_CO-3 and 5), 3.80 (3H, s, H_3_CO-4), 3.77 (6H, s, H_3_CO-3' and 5') 3.31 (2H, d, *J* = 6.5Hz, H-7'), 3.10 (1H, dd, *J* = 13.6 Hz, and 5.3 Hz , H-7a), 2.72 (1H, dd, *J* = 13.6 Hz and 7.6 Hz, H-7b), 1.20 (3H, d, *J* = 6.2 Hz, H-CH_3_-9), 0.93 (1H, dd, *J* = 6.5 and 1.0 Hz, H-9'); ^13^C-NMR (CDCl_3_, 125 MHz): δ_C_ 153.64 (C-3 and 5), 152.85 (C-1'), 137.24 (C-8'), 136.33 (C-4), 135.49 (C-4'), 134.89 (C-1), 134.35 (C-1'), 115.94 (C-9'), 106.58 (C-2 and 6), 105.94 (C-2' and 6'), 79.66 (C-8), 60.82 (C- H_3_CO-4), 56.08 (OCH2O-C), 56.17 (C- H_3_CO-3 and 5), 56.01 (C- H_3_CO-3' and 5'), 43.66 (C-7), 40.51 (C-7') and 19,76 (C- CH_3_-9). 

*3,5,7,3',4'-Pentahydroxyflavone* (**15**). Yellow crystals, mp 313–314 °C. ^1^H-NMR (CD_3_OD, 500 MHz) δ_H_: 6.38 (1H, d, *J* = 2.0 Hz, H-8), 6.17 (1H, d, *J* = 2.0 Hz, H-6), 7.62 (1H, dd, *J* = 8.5 and 2.5 Hz, H-6'), 6.88 (1H, d, *J* = 8.0 Hz, H-5'), 7.72 (1H, d, *J* = 2.5 Hz, H-2'). ^13^C-NMR (CD_3_OD, 125 MHz) δ_C_: 148.01 (C-2), 137.23 (C-3), 177.36 (C-4), 162.50 (C-5), 99.27 (C-6), 165.62 (C-7), 94.43 (C-8), 158.24 (C-9), 104.51 (C-10), 124.16 (C-1'), 116.23 (C-2'), 146.21 (C-3'), 148.76 (C-4), 116.01 (C-5'), 121.68 (C-6').

*5,7-Dihydroxy-4’-methoxyflavone* (**16**). Yellow crystals, m.p. 261–262 °C. ^1^H-NMR (D_­_MSO-d_6_, 200 MHz) δ_H_: 6.86 (s,1H, H-3), 6.20 (1H, d, *J* = 2.1 Hz, H-6), 6.50 (1H, d, *J* = 2.1 Hz, H-8), 8.03 (2H, d, *J* = 9.0 Hz, H-2'/H-6'), 7.10 (2H, d, *J* = 9.0 Hz, H-3'/H-5'), 3.85 (s, 3H, OCH_3_, H-4'). ^13^C-NMR (DMSO-d_6_, 50 MHz) δ_C_: 164.34 (C-2), 103.38 (C-3), 181.86 (C-4), 161.52 (C-5), 98.99 (C-6), 163.38 (C-7), 94.13 (C-8), 157.42 (C-9), 103.81 (C-10), 122.87 (C-1'), 128.40 (C-2'/C-6'), 114.67 (C-3'/C-5'), 162.38 (C-4), 55.64 (OCH3 C-4').

*5,8-Dihydroxy-7,4'-dimethoxyflavone* (**17**). Yellow powder. mp 265–266 °C. ^1^H-NMR (DMSO-d_6_, 500 MHz) δ_H_: 6.84 (s,1H, H-3), 6.53 (s, 1H, H-6), 8.09 (2H, dd, *J* = 7.0 and 2,0 Hz, H-2'/H-6'), 7.12 (2H, dd, *J* = 7.0 and 2.0 Hz, H-3'/H-5'), 3.88 (s, 3H, OCH_3_ H-7), 3.85 (s, 3H, OCH_3_ H-4') and 12.41 (s, 1H, OH-5). ^13^C-NMR (DMSO-d6, 125 MHz) δ_C_: 163.56 (C-2), 103.03 (C-3), 182.41 (C-4), 153.08 (C-5), 95.73 (C-6), 154.39 (C-7), 126.26 (C-8), 144.49 (C-9), 103.90 (C-10), 123.00 (C-1'), 128.51 (C-2'/C-6'), 114.59 (C-3'/C-5'), 162.41 (C-4'), 56.37 (OCH_3_ C-7) and 55.58 (OCH_3_ C-4').

*Kaempferol* *3-O-β-d-glucosyl-6''-α-L-ramnopyranoside* (**18**). Yellow powder; mp 220–221 °C. ^1^H-NMR (CD_3_OD, 500 MHz): δ_H_ 8.08 (2H, d, *J* = 8.6 Hz, H-2', 6'), 6.92 (2H, d, *J* = 8.6 Hz, H-3', 5'), 6.42 (1H, s, H-8), 6.23 (1H, s, H-6), 5.15 (1H, d, *J* = 7.5 Hz, H-’’), 3.30 (1H, d, *J* = 4.9 Hz, H-2'', 4.55 (1H, s, H-1'''), 3.84 (2H, d, *J* = 9.0 Hz, H-6'') 3.47 (3H, m, H-3'',4'',5''), 3.68 (1H, d, *J* = 7.1 Hz, H-2'''), 3.55 (1H, d, *J* = 3.3 Hz, H-3'''), 3.36 (1H, d, *J* = 5.4 Hz, H-4'''), 3.29 (1H, d, *J* = 5.4 Hz, H-4'''), 1.16 (3H, d, *J* = 6.2 Hz, H-6'''). ^13^C-NMR (CD_3_OD, 125 MHz): δ_C_ 159.36 (C-2), 135.51 (C-3), 179.35 (C-4), 162.92 (C-5), 100.02 (C-6), 166.15 (C-7), 94.96 (C-8), 158.24 (C-9), 105.59 (C-10), 122.73 (C-1'), 132.35 (C-2', C-6'), 116.12 (C-3', C-5'), 161.44 (C-4'), 104.64 (C-1''), 71.43 (C-2'', 78.14 (C-3''), 75,76 (C-4''), 69.71 (C-5'', 68.57 (C-6'', 102.40 (C-1'''), 72.07 (C-2'''), 72.30 (C-3'''), 77.18 (C-4'''), 73.90 (C-5'''), 17.90 (C-6''').

### 3.4. Antimicrobial Activity Experiments

The microorganisms used througout the tests were: *S. aureus* (ATCC 6538), *S. aureus* (ATCC 25923), *S. epidermidis* (ATCC 12228), *Bacillus subtilis* (ATCC 6633), *P. aeruginosa* (ATCC 25853), *P. aeruginosa* (ATCC 9027), *E. coli* (Classical C), *E. coli* (ATCC 18739), *E. coli* (ATCC 8733), *Shigella flexineri* (LM 412), *C. albicans* (ATCC 90028), *C. albicans* (ATCC 76,615), *C. albicans* (LM 142V), *C. albicans* (JCB 12), *C. tropicalis* (ATCC 13,803), *C. tropicalis* (LM 028), *C. krusei* (ATCC 6258), *C. krusei* (12 LM), *C guilliermondii* (LM 2101) and *C. guilliermondii* (LM 011). The strains were acquired from the collection of the Mycology Laboratory (LM), Institute of Biomedical Sciences, University of São Paulo (ICB-USP/SP) and the Oswaldo Cruz Foundation-FIOCRUZ (Rio de Janeiro). The stock bacterial strains were maintained in Muller Hinton agar (AMH) and the yeast on Sabouraud dextrose agar (SDA) under refrigeration (8 °C). The inoculum of microorganisms was prepared and standardized in sterilized saline (0.85%), containing Tween 80 (1%). Turbidity was visually compared and adjusted to the range of 0.5 tube McFarland, which corresponds to an inoculum of approximately 10^6^ CFU/mL (CFU/mL) [37,38,39]. The MIC determination was made by the microdilution method using 96 well plates (INLAB/Brazilian Industry) as protocol [39,40]. In each well of the plate was added 100 µL of liquid medium CSD or HCM doubly concentrated. Then was added 100 µL of each compound in the cavities of the first row of the plate, and by serial dilution concentrations were obtained 300 µg/mL to 9 µg/mL. Then was added 10 µL of the inoculum of microorganisms into the wells. Controls were: Tween 80 (10% in distilled water), chloramphenicol microbial growth control (30 µg/mL, Sigma-Aldrich^®^) for the bacteria and ketoconazole (50 µg/mL, Sigma-Aldrich^®^) for yeasts.

The assay was performed in duplicate and incubated at 35 °C/24 h. After the incubation time was added 20 µL of resazurin sodium 0.01% (w/v) (Sigma-Aldrich^®^) recognized as an indicator of oxidation-reduction colorimetric for bacteria. And in parallel, 20 μL of triphenyltetrazolium chloride (TTC) 1% (Sigma-Aldrich^®^), a colorimetric indicator for the redox yeast [41,42,43]. The assay was incubated at 35 °C. The reading of the test was performed by viewing the color change from blue to pink cavities tests of bactéria, and colorless to pink tests of yeast, indicating growth of the microorganism. MIC for each product was defined as the lowest concentration able to inhibit fungal growth visually, checked by the permanence of the color of the growth indicator.

Biological properties of the compounds were considered either active or non-active according to the criteria reported [31,32,33,34], in which natural compounds showing MIC between 50 and 500 μg/mL are classified as possessing strong antimicrobial activity, those with MIC from 500 to 1,500 μg/mL possess moderate activity and those with MIC larger than 1,500 μg/mL are considered with weak antimicrobial activity.

## 4. Conclusions

The chemical study of the crude extracts from *C. exaltata*, resulted in nineteen compounds: two mixtures of steroids **3a**, **3b**, **4a** and **4b**, four neolignans **1**, **2**, **13** and **14**, four porphyrins **5-8**, two phenolic acids **9** and **10**, two coumarins **11** and **12** and five flavonoids **15**–**19**. For the first time compounds **4a/4b**, **7**, **11**, **12**, **13**, **14**, **16**, **19** were isolated in Boraginaceae family, compounds **1**, **2**, **5**, **6**, **8**, **9** and**17** in genus *Cordia* and compounds **10**, **15** and **18** in species *C. exaltata*. Compounds **1** and **2** showed significant antimicrobial activity against bacteria and yeasts, whereas compounds **13** and **14** did not show a relevant effect.
